# Factors associated with multiple barriers to access to primary care: an international analysis

**DOI:** 10.1186/s12939-018-0740-1

**Published:** 2018-02-20

**Authors:** L. Corscadden, J. F. Levesque, V. Lewis, E. Strumpf, M. Breton, G. Russell

**Affiliations:** 1 Australian Institute of Tropical Health and Medicine, James Cook University, Townsville, QLD 4812 Australia; 2 Bureau of Health Information, Level 11, 67 Albert Avenue, Chatswood, NSW 2067 Australia; 3 Centre for Primary Health Care and Equity, University of New South Wales, Sydney, NSW 2052 Australia; 4 Australian Institute for Primary Care & Ageing, La Trobe University, Melbourne, VIC 3068 Australia; 5 Department of Economics and Department of Epidemiology, Biostatistics, and Occupational Health, McGill University, 855 Sherbrooke St. West, Montreal, QC H3A 2T7 Canada; 6 Department of community health, University of Sherbrooke, 150 Place Charles LeMoyne, Longueil, Québec, J4K 0A8 Canada; 7 General Practice Research, School of Primary and Allied Health Care, Monash University, 270 Ferntree Gull Rd Notting Hill, Melbourne, VIC 3168 Australia

**Keywords:** Primary care, Accessibility of healthcare services, Vulnerable groups, Mental health, Healthcare disparities

## Abstract

**Background:**

Disparities in access to primary care (PC) have been demonstrated within and between health systems. However, few studies have assessed the factors associated with multiple barriers to access occurring along the care-seeking process in different healthcare systems.

**Methods:**

In this secondary analysis of the 2016 Commonwealth Fund International Health Policy Survey of Adults, access was represented through participant responses to questions relating to access barriers either before or after reaching the PC practice in 11 countries (Australia, Canada, France, Germany, Norway, the Netherlands, New Zealand, Sweden, Switzerland, the United Kingdom, and United States). The number of respondents in each country ranged from 1000 to 7000 and the response rates ranged from 11% to 47%. We used multivariable logistic regression models within each of eleven countries to identify disparities in response to the access barriers by age, sex, immigrant status, income and the presence of chronic conditions.

**Results:**

Overall, one in five adults (21%) experienced multiple barriers before reaching PC practices. After reaching care, an average of 16% of adults had two or more barriers. There was a sixfold difference between nations in the experience of these barriers to access. Vulnerable groups experiencing multiple barriers were relatively consistent across countries. People with lower income were more likely to experience multiple barriers, particularly before reaching primary care practices. Respondents with mental health problems and those born outside the country displayed substantial vulnerability in terms of barriers after reaching care.

**Conclusion:**

A greater understanding of the multiple barriers to access to PC across the stages of the care-seeking process may help to inform planning and performance monitoring of disparities in access. Variation across countries may reveal organisational and system drivers of access, and inform efforts to improve access to PC for vulnerable groups. The cumulative nature of these barriers remains to be assessed.

**Electronic supplementary material:**

The online version of this article (10.1186/s12939-018-0740-1) contains supplementary material, which is available to authorized users.

## Background

Improving access to primary care (PC) is a goal of most healthcare systems. Disparities in access to care have been shown to exist between and within countries [1–5]. These disparities in access to PC in turn contribute to disparities in health, while improving access for vulnerable groups helps to reduce gaps in health outcomes [6, 7].

There are various ways to conceptualise access. From a patient perspective, access to care has been conceptualized as a process from perceiving a need for care and seeking care, to reaching and obtaining care and benefiting from the services received [8]. Reasons for unmet needs for healthcare have been demonstrated to exist at many stages, including in areas of availability, affordability and acceptability both before and after physically reaching a provider [9, 10].

From an empirical perspective, disparities in access to PC have been documented across a range of measures and vulnerable groups, with some consistencies and areas of divergence across countries. Foregoing care due to cost, difficulties with after-hours primary care, and timely access to PC appointments are more commonly experienced by people in lower income groups [5, 11–13]. Racial minorities and immigrants have lower rates of affiliation with a regular care provider and more unmet health needs, with disparities more pronounced in the United States than in Canada [14]. In many countries, people with multiple chronic conditions are more likely to have difficulties accessing after-hours care, and report having to wait several days to get an appointment when sick, compared to people with no conditions [5]. People with chronic conditions, particularly people with mental health conditions, were found to be more likely to forego care due to cost and have higher out-of-pocket healthcare costs than people with no chronic conditions [15].

When barriers to access to care accumulate, there are impacts upon healthcare use patterns. For example, studies have found a higher number of barriers to access were associated with more intensive use of emergency care in general [16] and for primary care reasons [17]. There is limited research investigating disparities in multiple barriers to access to PC and how patterns differ across countries. Therefore, this secondary data of an international survey systematically examines which population groups were more likely to experience multiple barriers to accessing PC across a range of measures, population groups and countries.

## Methods

We used the 2016 Commonwealth Fund International Health Policy Survey of adults aged 18 years and over in 11 countries: Australia, Canada, Germany, France, Netherlands, New Zealand, Norway, Sweden, Switzerland, the United Kingdom and the United States. Analyses were weighted so that the estimates were representative of the age, sex, regional and education profile of adults in each country. The number of respondents ranged between countries from 1000 to 7000 adults (Additional file 1: Appendix 1).

Our choice of access measures in this analysis was based on the conceptual model proposed by Levesque et al. [8] and followed an iterative prioritising process based on local innovation partnerships input described elsewhere [18] with additional considerations from key literature [17]. Responses were dichotomised (where applicable, responses of ‘sometimes, rarely and never’ were categorised as no, and ‘always and often’ grouped as yes). Next, measures were grouped into barriers experienced: before reaching a PC provider (no regular care provider; difficulties in accessing after-hours; difficulties in getting timely appointment or response to call; skipping tests, medication, or care due to cost) and after reaching a PC provider (regular care provider did not: listen carefully; know medical history; coordinate care, or spend enough time). The number of barriers experienced before and after reaching care for each person was calculated.

We considered vulnerable groups who may be more likely to face barriers to access to care as being those participants with chronic conditions, lower income, females, people over 65, and those not born in the country where they reside. In terms of chronic conditions, respondents were categorised into three groups: 1) people with no conditions; 2) those who said they had been diagnosed with a physical condition (including joint pain or arthritis, asthma or chronic lung disease, cancer, diabetes, heart disease, hypertension, high blood pressure, or stroke); and 3) those who said they were told by a doctor they had a mental health condition (regardless of the fact that they may have had other physical conditions).

For each country, the percentage of each vulnerable group, access barrier and combination of access barriers by group was calculated as well as a country average. Multivariable logistic regression models were run using SAS/STAT software, Version 9.3 (Copyright © 2005 SAS Institute Inc.) for each of the 11 countries to assess the likelihood of experiencing barriers to access to PC, adjusting for age, sex, income, chronic conditions, immigrant status as well as hospitalisation in the previous two years as a proxy for more intensive health service use. Our primary outcomes of interest are experiencing multiple barriers: 1) before; and 2) after reaching PC. As secondary outcomes, we considered each access barrier individually, calculating the number of times each vulnerable group was significantly more likely to experience barriers than a corresponding reference group was calculated across all countries (significant adjusted odds ratio where *p* < 0.05). The number of times each population group was significantly more likely to face barriers for each country was also summarised.

## Results

One fifth of adults (21%) on average reported experiencing two or more barriers before reaching PC (ranging from 6% to 38% across countries). After reaching PC, among adults who had a regular care provider, 16% reported experiencing two or more barriers (ranging from 5% to 30% across countries) (Table 1).

**Table 1:** Population characteristics and barriers to access: average percentage and range across countries

		Country average (%)	Range	Australia	Canada	New Zealand	United Kingdom	United States	Germany	Netherlands	France	Norway	Sweden	Switzerland
Independent variables - population characteristics														
Age	65 years and over	21	(17, 25)	17	19	19	22	19	25	22	25	21	25	22
Sex	Female	51	(50, 53)	50	52	53	51	52	51	51	52	50	50	51
Income	Below-average	38	(24, 50)	40	39	31	29	40	38	24	47	41	38	50
Chronic conditions	Mental health condition(s) w/wo physical	13	(4, 23)	13	20	13	11	23	9	8	4	16	20	13
	Physical health condition(s)	35	(29, 39)	29	39	34	31	39	32	32	36	39	37	35
Immigrant status	Not born in the country	17	(70, 93)	23	18	19	12	15	17	8	21	7	14	30
Hospitalization	Hospitalized overnight	18	(14, 30)	16	14	17	16	17	14	18	30	20	18	23
Dependent variables - Barriers to access to PC														
Before reaching PC	No doctor	15	(1, 58)	14	15	11	19	23	2	1	1	5	58	15
	^a^Have a regular place of care	95	(92, 100)	94	93	96	94	88	99	100	99	98	92	90
	After-hours access very difficult	24	(6, 42)	17	37	14	25	28	35	6	17	17	42	22
	Over five days to get appointment	18	(4, 31)	8	31	4	18	22	27	6	18	27	27	10
	No timely response to call	19	(12, 33)	14	33	17	21	28	13	13	14	22	24	12
	Skipped test or medication due to cost	11	(4, 28)	11	14	11	4	28	7	7	13	6	8	17
	Skipped GP consult due to cost	9	(3, 22)	9	6	14	4	22	3	3	9	5	4	20
	Any barrier before reaching PC	53	(25, 80)	42	68	40	52	67	66	25	47	48	80	50
After reaching PC	Care not coordinated	28	(18, 40)	19	22	19	24	28	40	25	37	34	40	18
	GP did not spend enough time	16	(6, 26)	8	20	12	15	19	14	6	16	21	26	13
	Medical history not known	16	(4, 29)	14	15	13	12	17	11	4	24	19	29	17
	Unclear explanations	12	(4, 27)	7	11	11	10	10	16	4	27	16	17	9
	Any barrier after reaching PC	38	(24, 58)	29	37	29	32	39	47	24	58	42	48	33
Multiple barriers	Two or more barriers before reaching PC	21	(6, 38)	15	33	17	18	38	15	6	18	17	36	19
	Two or more barriers after reaching PC	16	(5, 30)	10	17	13	14	18	16	5	30	20	24	12

^a^Note: All questions grouped regarding care after reaching PC were asked of people with a regular GP or place, therefore up to 8% of respondents across countries were excluded from answering the after reaching PC questions

**Fig. 1:**
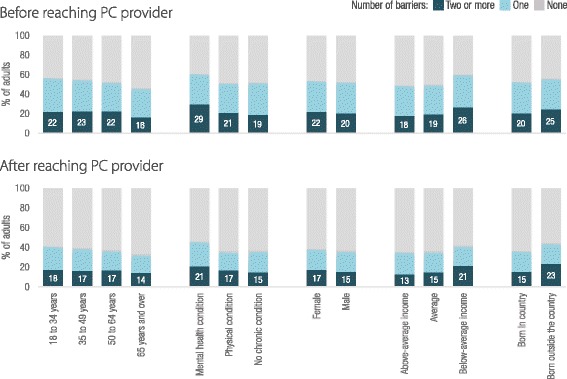
Percentage of adults experiencing multiple barriers to access both before and after reaching PC, average of countries by population characteristics. Note: Descriptive results based on unadjusted country averages, full results available in the technical appendix

**Fig. 2:**
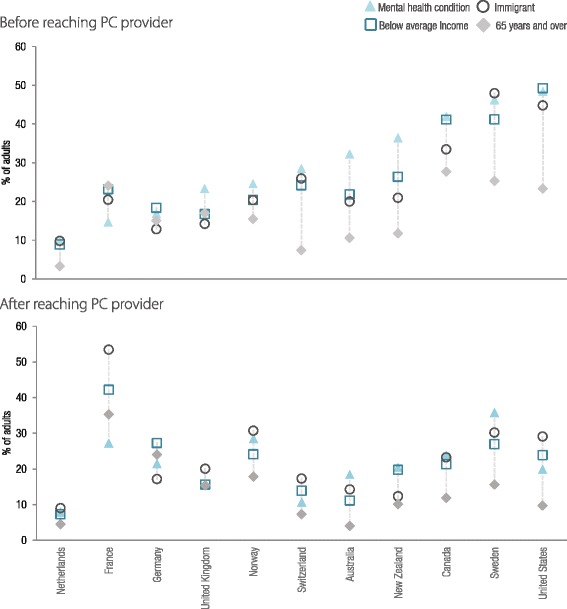
Percentage of adults reporting experience of multiple barriers to access both before and after reaching PC, by country and four selected vulnerable groups. Note: Descriptive results based on weighted prevalence within countries by population group, sorted by the percentage of people with a mental health condition experiencing barriers before reaching PC. For variables with more than two categories, the group with the most pronounced barriers was selected

Figure 1 shows the average percentage of people experiencing multiple barriers by vulnerable group. People with mental health conditions, those below average income, and those born outside their country of residence were more likely to face multiple barriers, whereas seniors were less likely to experience any barriers to access PC. Country level results for these four vulnerable groups are shown in Fig. 2. Before reaching PC, people with mental health conditions were more likely to experience multiple barriers, particularly in Australia and NZ. After reaching PC, people who were not born in the country appeared more likely to experience barriers, particularly in Norway, France, the UK, Switzerland, and the US.

**Fig. 3:**
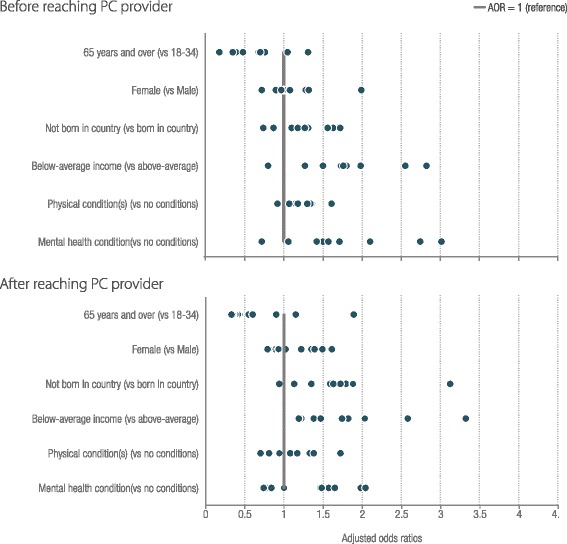
Adjusted odds ratios of multiple (two or more) barriers to access before and after reaching care across countries and population characteristics. Figure note: The lines represent the AOR = 1, adjusting for age, sex, immigrant status, income, chronic conditions, and being hospitalised. Each circle represents a country’s Adjusted Odds Ratio (AOR) from a full model estimating the outcomes of having multiple barriers to access before and after reaching PC. See the technical appendix for full results by country and access measure. Results are excluded if the country had fewer than 100 respondents with the selected barrier

Results of multivariable models estimating the odds of experiencing multiple (two or more) barriers before and after reaching care are summarised in Fig. 3 (full results are provided in the technical appendix showing country specific values and significance of each result). In most countries, people aged 65 years and over are less likely to experience multiple barriers than younger adults. People with below-average income were more likely to experience multiple barriers after reaching care with adjusted odds ratios (AOR) greater than one for all countries. Before reaching PC, people with below-average income, physical health conditions, and mental health conditions were more likely to experience barriers in all but one country (the UK, France and NZ for each of the vulnerabilities respectively). Being born outside the country of residence was associated with multiple barriers particularly after reaching PC for the US, Switzerland, France, Canada and Australia (AOR ranged from 1.59 to 3.12, *p* < 0.05). There were few significant differences by sex, however females were more likely to experience multiple barriers: before reaching care in NZ (AOR 1.99 p < 0.05), and after reaching care in France and Sweden (AOR. 1.49 and 1.39 respectively, the AOR for NZ was higher but not significant).

**Fig. 4:**
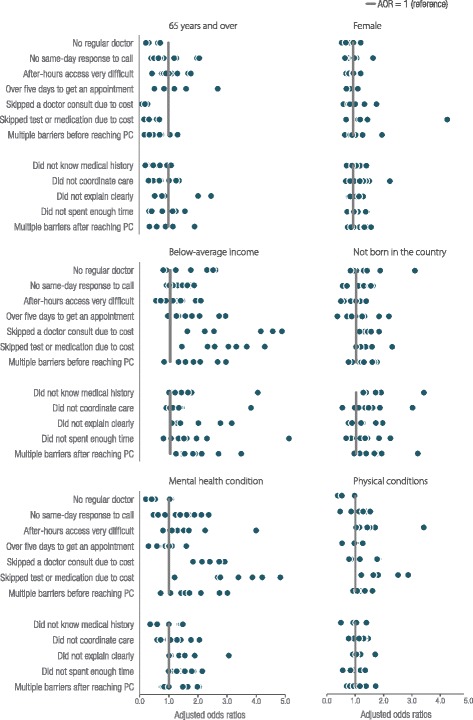
Adjusted odds ratios of all barriers to access, by countries and population characteristics, grouped by population. Figure note: Each circle represents a country’s AOR adjusting for age, sex, immigrant status, income, chronic conditions, and being hospitalised. The lines represent the AOR = 1. See appendix for full details on respondents per country for each access measure. Reference groups are: 65 and over vs 18 to 34 years, female vs male, not born in the country vs born in the country, below- average vs above-average income, presence of a physical condition(s) vs no conditions, and mental health condition (with or with out a physical one) vs no chronic conditions

The adjusted odds ratios for each access barrier individually as well as multiple barriers are provided in Fig. 4, to determine whether findings for multiple barriers are consistent across access measures. For almost all access measures older age was protective and below-average income was associated with a greater likelihood of barriers. However, in some countries older people experienced more barriers with timely access to GP care and receiving clear explanations.

For people with mental health conditions the extent of barriers in access to PC varied by country and type of access barrier. People with mental health conditions were consistently more likely to face affordability barriers; foregoing consultations and medication and tests due to cost across countries (AOR ranged from 1.20 to 4.83). However, they were less likely to say they had no regular care provider, or have long waits to see a GP compared to people with no mental health conditions in some countries.

**Fig. 5:**
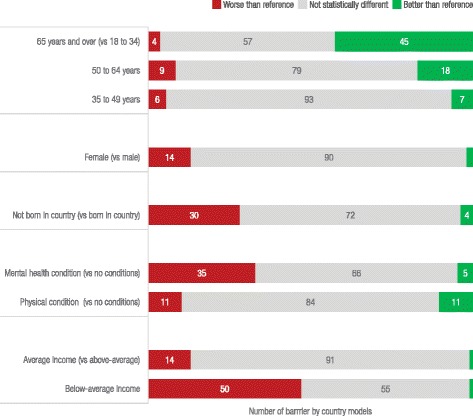
Number of significant differences for each population compared to reference group across countries and all barriers to access. Figure Note: There were 106 total comparisons where there were sufficient numbers of respondents out of a possible 132 models (11 countries * 12 access measures). Numbers of responses of ‘events’ or barriers to access for each access measure and country, and results by country are provided in the technical appendix. Results are based on full models adjusting for age, sex,immigrant status, income, chronic conditions, and being hospitalised in the past two years

A summary of the number of times each population group was significantly more likely to face barriers is presented in Fig. 5. For each country and access measure combination, we ran a model to estimate the odds of experiencing barriers for all vulnerable groups considered. In total, 106 models were generated where there were sufficient respondents. The most common difference was for people with below-average income, who were significantly more likely to experience access barriers than the above-average income group in 50 of 106 possible models. People with a mental health condition were more likely to experience barriers compared to people with no conditions in 35 models, and people born outside the country they reside in were more likely to experience barriers in 30 models. Older people were less likely than younger people to experience barriers in 45 of 106 models (see the technical appendix for complete results).

## Discussion

Access to PC spans from the identifiction of a health problem, to seeking care, to obtaining an appointment and reaching PC, and is affected by the characteristics of the interaction with the PC provider [8, 10]. Barriers to access to PC have been shown to vary by country and the measure of access [18]. This study goes further to assess which population groups are more likely to experience multiple access barriers, and summarise how patterns vary by population and measure of access.

Results presented in this secondary analysis of an international survey shows many people experience multiple barriers to care at different points in the pathway of accessing care. Further, certain population groups are disproportionately more likely to experience multiple barriers.

Factors associated with experiencing multiple barriers to access to care were generally consistent across countries. These factors included; below-average income, immigrant status, and chronic conditions, particularly mental health conditions. People with below-average income were more likely to experience barriers after reaching PC consistently across countries (AOR range across countries: 1.22 to 3.32). People with mental health conditions were more likely than people with no chronic conditions to experience multiple barriers before reaching PC, and statistically significant difference in 6 of 11 countries. Immigrants were more likely to experience multiple barriers, pariticuarly after reaching care (AOR ranged from 0.94 to 3.12). In contrast, age was protective, with people aged 65 and over less likely to experience barriers in most countries.

Findings regarding income and age were consistent with the literature. Systematic reporting of disparities across 21 measures of access in the United States showed lower income was a risk factor for all measures, with disparities by ethnicity also prevalent but less pronounced [1]. Similarly, we found the number of significant differences by income to be the most persistent disparity across countries. Findings from past Commonwealth Fund International Health Policy surveys have shown that low income was a signficant risk factor across most countries and older age was protective for most access measures [5].

There is broad interest in addressing barriers in access to care for a range of vulnerable groups [19, 20]. There are many possible insights into why barriers exist or how to address them. In terms of barriers faced by people with low income, qualitative research has suggested provider lack of understanding of living in poverty may lead to the development of inappropriate care plans that do not acknowledge and account for patients’ social circumstances [21]. Disparities in access to PC faced by people born outside the country they reside in may indicate issues around seeking care; however, results in this analysis suggested they also experienced disparities after reaching PC, which may reflect language and cultural differences between patients and providers. Finally, for people with mental health conditions, there may be additional factors at play such as the stigmatisation related to seeking care [22] as well as a lack of preparation of PC providers to deal with mental health issues [23].

As access is conceptualised from both provider and patient perspectives in the Levesque et al. model [8], we also consider factors related to both supply of, and demand for care to contribute to the reasons some groups experience multiple barriers to accessing care. In terms of demand, it has been suggested that people with mental health concerns are less likely to seek care, and interventions building trust in their physicians was a protective factor in care-seeking [24]. Provider preference or comfort in managing certain health conditions, particularly mental health, may also be a factor contributing to disparities some groups face. In a regular survey of primary care providers, GPs were asked if they felt their clinic was prepared to manage care for different population groups. Fewer than half of GPs in the 11 surveyed countries said they were well prepared to manage people with serious mental health conditions or substance use issues – consistently lower than perceptions of preparedness to manage multiple chronic conditions [23].

This study suggests that factors associated with barriers to access do not occur in isolation and may be clustered and multifactorial. Research considering multiple risk factors also suggests that clinical and social factors can also accumulate and interact to influence access to care [25]. This model recognises that factors associated with poorer access to PC are interconnected, such that people experiencing multiple vulnerability factors may have even greater barriers to PC. For example, developing mental health conditions can impact income, and having lower income can impact mental health [26]. Another model of vulnerability suggests a risk factor profile approach to understanding disparities in healthcare [27]. Research has shown that an increased number of risk factors is associated with a greater likelihood of unmet needs [28]. Future work could consider the possible cumulative effects of multiple factors on the experiences of barriers to access to PC.

### Limitations

There are limitations to this study that should be acknowledged. As it was a secondary analysis, the survey questions did not completely cover all five domains of the conceptual framework of access to care [8, 10]; therefore, we have considered a simplification of barriers before and after reaching PC. We have assumed the selected measures apply to primary care, rather than all healthcare. The survey also did not include information regarding ethnicity or language spoken for all countries. Futher, rurality and education measures could not be created to be comparable across countries. These population factors are also known to be associated with barriers to access to PC. Finally, the prevalence of people reporting they had mental health problems, ranged from 4% to 23% across countries and is likely to be underreported in some countries and through self-reported single item questions. For example, for Australia the prevalence of self-reported mental health problems was 13%, slightly lower than the 17.5% estimate from the National Health Survey [29].

There are data limitaitons associated with the survey that affect the comparability of disparity results across countries. First, there were different numbers of respondents and response rates for each country, therefore different power to detect significant differences. Our results reflect patterns across countries in the relationships and do not compare the size of disparities. We address issues of multiple comparisons in a descriptive manner by placing counts of significant differences, alongside descriptive bivariate patterns, as well as the size of odds ratios from multivariable models to identify consistant patterns in findings. Further, as analysis is based on survey data with wide margins of error (+/− 3–4%) [4] adjusted odds of 1.5 or more were generally significant, a magnitude which also appears meaningful from a face-validity perspective. Future work to understand the associations between population factors and access barriers over time in each country, as well as interactions between factors such as income and gender or health conditions may provide a fuller picture of patterns of disparities.

## Conclusions

There are many differences in the political and economic climates of the countries whose data were analysed; however, our findings show many consistent patterns in disparities of access to PC for various vulnerable groups, as well as some that are more pronounced in certain contexts. Further our findings demonstrate the cumulative nature of barriers preventing people from fully accessing PC. Considering the characteristics of population groups that are more likely to experience barriers to access and the reasons they might have issues seeking, reaching or fully accessing care may help reorient health services to address disparities in access to PC. Country and population group differences in disparities suggest inequities in access are amenable to a range of policy, organisational and educational responses to reduce them.

## Additional file

Additional file 1: Factors associated with multiple barriers to access to primary care - Technical Appendix. (DOCX 86.9 kb)

## Abbreviations

AOR: Adjusted Odds Ratio
GP: General Practitioner
IMPACT: Innovative Models Promoting Access to Care Transformation
NZ: New Zealand
PC: Primary care
UK: United Kingdom
US: United States
