# Supplementary material for: Factors associated with multiple barriers to access to primary care: an international analysis
Source: Int J Equity Health. 2018 Feb 20;17:28. doi: 10.1186/s12939-018-0740-1 (PMC5819269; doi:10.1186/s12939-018-0740-1)
Supplement: Supplementary file 1 — Factors associated with multiple barriers to access to primary care - Technical Appendix. (DOCX 86.9 kb) [file 12939_2018_740_MOESM1_ESM.docx]

**Appendix 1: Respondents and response rates by country and survey type**

|  | Landline | Cell phone | Total  (%) | Total |
| --- | --- | --- | --- | --- |
| Australia | 23.70% | 27.80% | 25.40% | 5,248 |
| Canada | 23.10% | 16.90% | 21.40% | 4,547 |
| France | 24.50% | 26.80% | 25.20% | 1,103 |
| Germany | 27.00% | 26.60% | 26.90% | 1,000 |
| Netherlands | 32.40% | 32.40% | 32.40% | 1,227 |
| New Zealand | 35.20% | 23.40% | 31.10% | 1,000 |
| Norway | 10.60% | 11.00% | 10.90% | 1,093 |
| Sweden | 17.60% | 16.40% | 16.90% | 7,124 |
| Switzerland | *86% of respondents by web* | | 46.90% | 1,520 |
| United Kingdom | 26.10% | 13.90% | 21.90% | 1,000 |
| United States | 19.40% | 17.20% | 18.10% | 2,001 |

Appendix 2: Percentage of adults reporting multiple barriers to access to care either before or after reaching PC, by population group and country

|  |  | | | **Percentage with multiple barriers before reaching PC (%)** | | | | | | | | | | | | | **Percentage with multiple barriers after reaching PC (%)** | | | | | | | | | | |  | |
| --- | --- | --- | --- | --- | --- | --- | --- | --- | --- | --- | --- | --- | --- | --- | --- | --- | --- | --- | --- | --- | --- | --- | --- | --- | --- | --- | --- | --- | --- |
|  |  | | Australia | | Canada | France | Germany | Netherlands | New Zealand | Norway | Sweden | Switzerland | United Kingdom | United States | **Average** | Australia | | Canada | France | Germany | Netherlands | New Zealand | Norway | Sweden | Switzerland | United Kingdom | United States | | **Average** |
| Age | | 18 to 34 years | 11 | | 30 | 18 | 11 | 7 | 20 | 19 | 40 | 26 | 21 | 42 | **22** | 10 | | 23 | 25 | 13 | 4 | 15 | 19 | 29 | 16 | 16 | 24 | | **18** |
|  | | 35 to 49 years | 17 | | 38 | 13 | 16 | 5 | 16 | 16 | 43 | 27 | 15 | 41 | **23** | 11 | | 19 | 26 | 12 | 4 | 10 | 24 | 29 | 17 | 13 | 18 | | **17** |
|  | | 50 to 64 years | 24 | | 37 | 16 | 17 | 6 | 17 | 16 | 38 | 15 | 20 | 41 | **22** | 12 | | 14 | 36 | 16 | 7 | 16 | 20 | 23 | 9 | 12 | 19 | | **17** |
|  | | 65 years and over | 11 | | 28 | 24 | 15 | 3 | 12 | 15 | 25 | 7 | 17 | 23 | **16** | 4 | | 12 | 35 | 24 | 5 | 10 | 18 | 16 | 7 | 15 | 10 | | **14** |
| Sex | | Female | 17 | | 34 | 18 | 13 | 6 | 21 | 19 | 38 | 20 | 17 | 38 | **22** | 11 | | 18 | 34 | 16 | 5 | 16 | 23 | 28 | 11 | 13 | 18 | | **17** |
|  | | Male | 13 | | 33 | 18 | 17 | 5 | 11 | 15 | 35 | 19 | 19 | 38 | **20** | 9 | | 16 | 26 | 16 | 5 | 10 | 18 | 20 | 14 | 15 | 19 | | **15** |
| Income | | Below-average | 22 | | 41 | 23 | 18 | 9 | 26 | 20 | 41 | 24 | 17 | 49 | **26** | 11 | | 21 | 42 | 27 | 7 | 20 | 24 | 27 | 14 | 16 | 24 | | **21** |
|  | | Average | 13 | | 32 | 14 | 12 | 4 | 15 | 12 | 37 | 15 | 21 | 38 | **19** | 11 | | 17 | 27 | 12 | 5 | 9 | 17 | 22 | 11 | 12 | 18 | | **15** |
|  | | Above-average | 12 | | 26 | 16 | 13 | 4 | 11 | 16 | 34 | 15 | 18 | 28 | **18** | 8 | | 13 | 15 | 11 | 3 | 12 | 18 | 23 | 10 | 11 | 14 | | **13** |
| Chronic Conditions | | Mental health condition | 32 | | 42 | 15 | 17 | 10 | 36 | 25 | 46 | 29 | 23 | 48 | **29** | 18 | | 24 | 27 | 22 | 8 | 20 | 29 | 36 | 11 | 15 | 20 | | **21** |
|  |  | Physical condition | 15 | | 34 | 21 | 17 | 7 | 13 | 16 | 32 | 18 | 20 | 35 | **21** | 6 | | 14 | 42 | 20 | 6 | 12 | 20 | 21 | 12 | 18 | 15 | | **17** |
|  |  | No chronic conditions | 11 | | 28 | 16 | 13 | 4 | 14 | 15 | 36 | 18 | 16 | 35 | **19** | 9 | | 16 | 24 | 14 | 4 | 12 | 18 | 21 | 13 | 11 | 21 | | **15** |
| Immigrant | | Not born in the country | 20 | | 33 | 20 | 13 | 10 | 21 | 20 | 48 | 26 | 14 | 45 | **25** | 14 | | 23 | 53 | 17 | 9 | 12 | 31 | 30 | 17 | 20 | 29 | | **23** |
|  | | Born in the country | 14 | | 33 | 17 | 15 | 5 | 16 | 16 | 35 | 17 | 19 | 36 | **20** | 9 | | 16 | 24 | 16 | 5 | 13 | 19 | 23 | 10 | 13 | 16 | | **15** |

**Appendix 3 :** Number of respondents for each of the barriers to access to PC, by country

|  | Australia | Canada | France | Germany | Netherlands | New Zealand | Norway | Sweden | Switzerland | United Kingdom | United States |
| --- | --- | --- | --- | --- | --- | --- | --- | --- | --- | --- | --- |
| No regular doctor | 521 | 520 | 11 | 18 | 17 | 111 | 40 | 3648 | 178 | 174 | 357 |
| No same-day response to call | 540 | 1146 | 144 | 113 | 133 | 142 | 192 | 1221 | 144 | 179 | 417 |
| After-hours access very difficult | 743 | 1484 | 160 | 310 | 54 | 104 | 118 | 1781 | 220 | 180 | 428 |
| Over five days to get an appointment | 439 | 1232 | 157 | 300 | 64 | 42 | 281 | 1601 | 163 | 158 | 342 |
| Skipped a doctor consult due to cost | 255 | 196 | 61 | 29 | 48 | 134 | 41 | 142 | 211 | 34 | 359 |
| Skipped a recommended treatment or medication | 364 | 476 | 111 | 59 | 81 | 102 | 54 | 324 | 181 | 39 | 498 |
| Did not know history | 586 | 530 | 199 | 99 | 47 | 96 | 170 | 1298 | 193 | 111 | 242 |
| Did not coordinate care | 724 | 734 | 311 | 333 | 230 | 148 | 227 | 1263 | 196 | 186 | 396 |
| Did not explain clearly | 202 | 386 | 274 | 159 | 45 | 80 | 143 | 845 | 94 | 88 | 143 |
| Did not spend enough time | 374 | 692 | 173 | 139 | 72 | 108 | 201 | 1358 | 164 | 146 | 287 |
| Multiple barriers before reaching PC | 650 | 1425 | 145 | 146 | 70 | 156 | 165 | 2355 | 259 | 175 | 657 |
| Multiple barriers after reaching PC | 392 | 598 | 266 | 154 | 62 | 109 | 198 | 1284 | 155 | 139 | 271 |
| Where number of events <100 models were not run  Total models run (Total=106 of 132) | 12 | 12 | 10 | 8 | 2 | 9 | 9 | 12 | 11 | 9 | 12 |
|  | | | | | | | | | | | |
| Note: Results were excluded for a given country where the number of respondents experiencing barriers was less than 10 times the degrees of freedom, here we used a cut-off of 100. Analysis were not conducted for the country and access measure combinations with fewer than 100 responses. The power to detect significant differences is greater in countries such as Australia, Canada and Sweden which have larger numbers of respondents. | | | | | | | | | | | |

Appendix 4. Adjusted odds ratios of experiencing barriers to PC, by country (part 1 of 2)

|  | **Australia** | **Canada** | **France** | **Germany** | **Netherlands** | **NZ** | **Norway** | **Sweden** | **Switzerland** | **UK** | **US** |
| --- | --- | --- | --- | --- | --- | --- | --- | --- | --- | --- | --- |
| **No regular doctor** |  |  |  |  |  |  |  |  |  |  |  |
| 35 to 49 years (vs 18 to 34) | 0.96 | 0.55 |  |  |  | 0.56 |  | 0.59 | 0.84 | 0.50 | 0.63 |
| 50 to 64 years | 0.47 | 0.38 |  |  |  | 0.57 |  | 0.45 | 0.5 | 0.74 | 0.38 |
| 65 years and over | 0.34 | 0.29 |  |  |  | 0.64 |  | 0.30 | 0.33 | 0.72 | 0.23 |
| Mental health condition (vs no condition) | 1.08 | 0.28 |  |  |  | 0.53 |  | 0.36 | 0.21 | 1.03 | 0.41 |
| Physical condition(s) | 0.49 | 0.36 |  |  |  | 0.56 |  | 0.38 | 0.53 | 0.98 | 0.44 |
| Female (vs male) | 0.95 | 0.61 |  |  |  | 0.89 |  | 0.75 | 1.01 | 1.26 | 0.75 |
| Average income (vs above-average) | 0.70 | 1.56 |  |  |  | 1.55 |  | 1.00 | 0.65 | 1.15 | 1.61 |
| Below-average income | 1.67 | 2.48 |  |  |  | 2.20 |  | 1.18 | 0.87 | 0.77 | 2.40 |
| Not born in country (vs born in country) | 3.02 | 1.01 |  |  |  | 0.92 |  | 1.26 | 1.83 | 0.81 | 1.38 |
| **No same-day response to call** |  |  |  |  |  |  |  |  |  |  |  |
| 35 to 49 years (vs 18 to 34) | 1.06 | 1.70 | 0.90 | 1.31 | 0.99 | 1.12 | 1.26 | 1.30 | 1.33 | 0.72 | 0.79 |
| 50 to 64 years | 1.62 | 1.40 | 1.17 | 1.29 | 0.6 | 1.19 | 0.93 | 1.18 | 0.77 | 0.78 | 0.90 |
| 65 years and over | 0.86 | 0.82 | 1.97 | 2.05 | 0.41 | 1.27 | 1.19 | 0.84 | 0.51 | 0.72 | 0.66 |
| Mental health condition (vs no condition) | 1.69 | 1.24 | 0.47 | 0.63 | 0.87 | 1.61 | 2.37 | 1.23 | 2.12 | 1.88 | 1.39 |
| Physical condition(s) | 1.17 | 1.17 | 0.47 | 0.47 | 1.39 | 0.86 | 1.38 | 1.14 | 1.28 | 1.53 | 1.03 |
| Female (vs male) | 1.14 | 1.04 | 0.91 | 1.03 | 0.74 | 1.68 | 0.84 | 0.99 | 0.72 | 1.12 | 1.04 |
| Average income (vs above-average) | 0.93 | 1.06 | 0.83 | 0.9 | 0.56 | 0.93 | 1.05 | 1.00 | 1.12 | 0.58 | 1.39 |
| Below-average income | 0.92 | 1.21 | 0.88 | 1.65 | 1.05 | 1.24 | 1.09 | 1.4 | 1.78 | 0.97 | 1.56 |
| Not born in country (vs born in country) | 1.35 | 1.08 | 0.54 | 1.25 | 1.08 | 1.27 | 1.56 | 1.58 | 1.46 | 0.69 | 1.51 |
| **After-hours access very difficult** |  |  |  |  |  |  |  |  |  |  |  |
| 35 to 49 years (vs 18 to 34) | 0.99 | 1.67 | 0.95 | 0.68 |  | 1.31 | 0.91 | 1.67 | 1.00 | 1.23 | 1.23 |
| 50 to 64 years | 2.01 | 1.87 | 0.56 | 0.72 |  | 1.17 | 0.79 | 1.57 | 0.90 | 1.13 | 1.46 |
| 65 years and over | 1.67 | 1.76 | 1.12 | 0.44 |  | 0.83 | 0.77 | 1.30 | 0.84 | 1.02 | 0.92 |
| Mental health condition (vs no condition) | 1.04 | 1.29 | 0.80 | 4.00 |  | 2.25 | 1.65 | 1.11 | 1.60 | 1.51 | 1.69 |
| Physical condition(s) | 1.00 | 1.45 | 1.70 | 3.42 |  | 1.51 | 1.15 | 1.02 | 1.03 | 1.42 | 1.37 |
| Female (vs male) | 1.28 | 1.26 | 0.76 | 0.86 |  | 0.99 | 1.29 | 1.01 | 1.26 | 1.28 | 1.26 |
| Average income (vs above-average) | 0.95 | 1.09 | 0.70 | 0.77 |  | 1.23 | 0.71 | 1.15 | 0.90 | 0.63 | 1.36 |
| Below-average income | 1.24 | 1.40 | 1.07 | 0.53 |  | 1.83 | 1.09 | 1.34 | 0.86 | 0.69 | 2.00 |
| Not born in country (vs born in country) | 0.82 | 0.68 | 0.78 | 0.58 |  | 0.82 | 1.20 | 1.14 | 1.35 | 0.48 | 1.00 |
| **Over five days to get an appointment** |  |  |  |  |  |  |  |  |  |  |  |
| 35 to 49 years (vs 18 to 34) | 0.95 | 1.44 | 0.45 | 1.58 |  |  | 1.34 | 0.67 | 0.94 | 0.76 | 0.95 |
| 50 to 64 years | 2.44 | 1.57 | 0.89 | 1.04 |  |  | 2.25 | 0.81 | 0.67 | 1.22 | 1.65 |
| 65 years and over | 1.27 | 1.24 | 1.6 | 0.52 |  |  | 2.68 | 0.89 | 0.85 | 1.18 | 1.20 |
| Mental health condition (vs no condition) | 0.88 | 0.99 | 0.64 | 0.3 |  |  | 0.59 | 1.6 | 1.11 | 1.01 | 1.01 |
| Physical condition(s) | 1.04 | 1.28 | 0.52 | 1.21 |  |  | 0.53 | 1.21 | 0.97 | 1.26 | 0.78 |
| Female (vs male) | 0.75 | 1.04 | 1.13 | 0.94 |  |  | 0.89 | 1.17 | 0.74 | 1.16 | 0.77 |
| Average income (vs above-average) | 1.08 | 1.23 | 1.02 | 1.45 |  |  | 0.65 | 0.95 | 1.21 | 0.87 | 1.7 |
| Below-average income | 1.96 | 1.74 | 1.71 | 2.81 |  |  | 1.49 | 1.27 | 1.2 | 0.92 | 2.6 |
| Not born in country (vs born in country) | 1.32 | 0.98 | 1.79 | 0.36 |  |  | 0.69 | 1.29 | 1.21 | 0.85 | 2.13 |
| **Skipped a doctor consult due to cost** |  |  |  |  |  |  |  |  |  |  |  |
| 35 to 49 years (vs 18 to 34) | 2.26 | 1.25 |  |  |  | 0.80 |  | 0.68 | 0.87 |  | 1.03 |
| 50 to 64 years | 1.50 | 0.55 |  |  |  | 0.44 |  | 0.89 | 0.36 |  | 0.66 |
| 65 years and over | 0.31 | 0.29 |  |  |  | 0.14 |  | 0.16 | 0.10 |  | 0.20 |
| Mental health condition (vs no condition) | 2.92 | 2.41 |  |  |  | 2.73 |  | 2.12 | 2.14 |  | 1.83 |
| Physical condition(s) | 0.85 | 1.81 |  |  |  | 0.78 |  | 1.16 | 1.77 |  | 1.57 |
| Female (vs male) | 1.39 | 0.67 |  |  |  | 1.80 |  | 1.07 | 0.83 |  | 0.89 |
| Average income (vs above-average) | 1.35 | 1.93 |  |  |  | 1.12 |  | 1.22 | 1.16 |  | 1.48 |
| Below-average income | 1.56 | 4.35 |  |  |  | 3.96 |  | 4.65 | 2.43 |  | 2.13 |
| Not born in country (vs born in country) | 1.41 | 1.79 |  |  |  | 1.28 |  | 1.40 | 1.52 |  | 1.12 |
| **Skipped a recommended test/treatment/medication due to cost** | | |  |  |  |  |  |  |  |  |  |
| 35 to 49 years (vs 18 to 34) | 1.71 | 1.06 | 0.97 |  |  | 0.73 |  | 0.86 | 1.49 |  | 1.00 |
| 50 to 64 years | 1.79 | 0.60 | 0.77 |  |  | 0.56 |  | 0.79 | 0.78 |  | 0.83 |
| 65 years and over | 0.60 | 0.22 | 0.69 |  |  | 0.22 |  | 0.17 | 0.36 |  | 0.34 |
| Mental health condition (vs no condition) | 3.87 | 4.83 | 3.39 |  |  | 4.21 |  | 2.68 | 1.20 |  | 2.77 |
| Physical condition(s) | 1.18 | 2.87 | 2.51 |  |  | 1.81 |  | 1.64 | 1.22 |  | 2.00 |
| Female (vs male) | 1.47 | 1.16 | 0.76 |  |  | 4.22 |  | 1.49 | 1.17 |  | 1.24 |
| Average income (vs above-average) | 1.30 | 2.86 | 1.13 |  |  | 1.60 |  | 1.22 | 1.71 |  | 1.27 |
| Below-average income | 2.45 | 4.09 | 1.38 |  |  | 3.50 |  | 2.90 | 3.16 |  | 2.22 |
| Not born in country (vs born in country) | 1.36 | 1.56 | 1.33 |  |  | 1.55 |  | 2.24 | 1.16 |  | 1.00 |
|  | | | | | | | | | | | |

Note: *Darker shading indicates where all countries AOR are above 1, and lighted where AOR<1 (p<0.05). Missing values for countries indicate there were not sufficent responses with the barrier to access in that country to run the full model (n<100). Results are from full models within countries for each access barrier adjusting for the variables listed as well as hospitalisation in the past two years.*

Appendix 4. Adjusted odds ratios of experiencing barriers to PC, by country (part 2 of 2)

|  | **Australia** | **Canada** | **France** | **Germany** | **Netherlands** | **NZ** | **Norway** | **Sweden** | **Switzerland** | **UK** | **US** |
| --- | --- | --- | --- | --- | --- | --- | --- | --- | --- | --- | --- |
| **Did not know medical history** |  |  |  |  |  |  |  |  |  |  |  |
| 35 to 49 years (vs 18 to 34) | 1.48 | 0.62 | 1.13 |  |  |  | 0.89 | 1.23 | 1.20 | 1.17 | 0.71 |
| 50 to 64 years | 0.98 | 0.57 | 1.28 |  |  |  | 0.68 | 0.74 | 0.69 | 0.96 | 0.72 |
| 65 years and over | 0.29 | 0.54 | 0.95 |  |  |  | 0.71 | 0.43 | 0.21 | 1.09 | 0.48 |
| Mental health condition (vs no conditions) | 1.36 | 1.37 | 0.36 |  |  |  | 1.45 | 1.42 | 0.60 | 1.48 | 1.00 |
| Physical condition(s) | 0.50 | 0.99 | 1.39 |  |  |  | 0.94 | 1.02 | 0.93 | 1.04 | 0.8 |
| Female (vs male) | 0.92 | 1.04 | 1.23 |  |  |  | 1.24 | 1.45 | 1.10 | 0.78 | 0.97 |
| Average income (vs above-average) | 1.42 | 1.43 | 1.85 |  |  |  | 0.79 | 0.87 | 0.77 | 1.34 | 1.56 |
| Below-average income | 1.36 | 1.70 | 3.86 |  |  |  | 1.58 | 1.05 | 0.96 | 1.16 | 1.58 |
| Not born in country (vs born in country) | 1.69 | 1.67 | 3.33 |  |  |  | 1.86 | 1.28 | 1.35 | 1.25 | 1.67 |
| **Did not coordinate care** |  |  |  |  |  |  |  |  |  |  |  |
| 35 to 49 years (vs 18 to 34) | 1.25 | 0.93 | 0.99 | 0.71 | 0.96 | 1.01 | 1.05 | 0.99 | 1.68 | 0.73 | 0.89 |
| 50 to 64 years | 1.28 | 0.64 | 1.43 | 0.74 | 1.21 | 1.4 | 0.57 | 0.61 | 1.05 | 0.73 | 0.61 |
| 65 years and over | 0.69 | 0.56 | 1.02 | 0.37 | 0.74 | 1.36 | 0.48 | 0.48 | 1.26 | 0.69 | 0.32 |
| Mental health condition (vs no condition) | 0.96 | 1.02 | 0.60 | 1.76 | 1.45 | 1.06 | 2.05 | 1.41 | 0.72 | 1.28 | 1.06 |
| Physical condition(s) | 0.73 | 0.90 | 1.33 | 0.75 | 1.45 | 0.94 | 1.34 | 1.29 | 0.77 | 1.13 | 0.98 |
| Female (vs male) | 1.56 | 1.02 | 1.45 | 0.90 | 1.01 | 2.26 | 1.04 | 1.35 | 0.75 | 1.23 | 0.90 |
| Average income (vs above-average) | 0.82 | 0.90 | 1.83 | 0.60 | 0.83 | 0.65 | 0.76 | 0.91 | 0.61 | 0.84 | 1.29 |
| Below-average income | 1.15 | 1.38 | 3.64 | 1.25 | 0.87 | 0.99 | 1.08 | 1.12 | 1.33 | 1.08 | 1.28 |
| Not born in country (vs born in country) | 1.19 | 1.53 | 2.94 | 0.53 | 0.95 | 1.30 | 1.31 | 1.09 | 1.82 | 1.57 | 1.43 |
| **Did not explain clearly** |  |  |  |  |  |  |  |  |  |  |  |
| 35 to 49 years (vs 18 to 34) | 0.85 | 1.08 | 1.43 | 1.66 |  |  | 1.17 | 1.08 |  |  | 0.55 |
| 50 to 64 years | 0.56 | 0.77 | 1.69 | 1.31 |  |  | 0.98 | 0.78 |  |  | 0.83 |
| 65 years and over | 0.55 | 0.87 | 2.01 | 2.45 |  |  | 0.78 | 0.49 |  |  | 0.53 |
| Mental health condition (vs no condition) | 3.06 | 1.57 | 1.35 | 1.07 |  |  | 1.89 | 1.55 |  |  | 1.01 |
| Physical condition(s) | 1.17 | 0.96 | 0.91 | 1.71 |  |  | 1.19 | 1.04 |  |  | 1.06 |
| Female (vs male) | 1.02 | 1.12 | 1.34 | 1.11 |  |  | 0.85 | 1.20 |  |  | 0.90. |
| Average income (vs above-average) | 1.18 | 1.39 | 1.25 | 2.05 |  |  | 1.54 | 1.20 |  |  | 2.00 |
| Below-average income | 1.29 | 1.23 | 1.92 | 3.01 |  |  | 1.05 | 1.33 |  |  | 2.64 |
| Not born in country (vs born in country) | 1.91 | 1.71 | 0.74 | 0.86 |  |  | 1.68 | 1.23 |  |  | 1.17 |
| **Did not spend enough time** |  |  |  |  |  |  |  |  |  |  |  |
| 35 to 49 years (vs 18 to 34) | 1.44 | 0.85 | 1.30 | 1.51 |  | 0.87 | 1.10 | 1.01 | 0.92 | 0.84 | 0.72 |
| 50 to 64 years | 0.72 | 0.63 | 1.14 | 1.59 |  | 1.38 | 1.07 | 0.78 | 0.53 | 0.66 | 0.88 |
| 65 years and over | 0.49 | 0.40 | 1.56 | 1.25 |  | 1.14 | 0.78 | 0.47 | 0.34 | 0.79 | 0.43 |
| Mental health condition (vs no condition) | 1.40 | 1.85 | 2.15 | 1.08 |  | 1.28 | 1.79 | 1.54 | 1.27 | 1.55 | 0.99 |
| Physical condition(s) | 0.56 | 1.14 | 1.29 | 0.95 |  | 0.94 | 0.83 | 1.19 | 1.36 | 1.35 | 0.87 |
| Female (vs male) | 0.85 | 0.80 | 1.02 | 1.13 |  | 1.47 | 1.17 | 1.43 | 0.99 | 1.04 | 1.03 |
| Average income (vs above-average) | 1.41 | 1.67 | 1.30 | 2.68 |  | 0.58 | 1.25 | 1.06 | 0.65 | 1.10 | 1.14 |
| Below-average income | 1.33 | 1.86 | 1.03 | 4.88 |  | 2.20 | 1.54 | 1.27 | 1.27 | 0.78 | 1.45 |
| Not born in country (vs born in country) | 2.18 | 1.25 | 0.83 | 1.03 |  | 0.66 | 1.39 | 1.81 | 1.15 | 1.31 | 1.79 |
| **Multiple barriers before reaching PC** | | | | |  |  |  |  |  |  |  |
| 35 to 49 years (vs 18 to 34) | 1.55 | 1.51 | 0.69 | 1.84 |  | 0.81 | 0.85 | 1.14 | 0.99 | 0.59 | 1.03 |
| 50 to 64 years | 2.00 | 1.31 | 0.83 | 1.63 |  | 0.71 | 0.81 | 0.91 | 0.45 | 0.89 | 0.88 |
| 65 years and over | 0.68 | 0.72 | 1.31 | 1.05 |  | 0.39 | 0.76 | 0.48 | 0.18 | 0.70 | 0.35 |
| Mental health condition (vs no condition) | 2.74 | 1.50 | 0.72 | 1.06 |  | 3.01 | 1.72 | 1.42 | 2.1 | 1.57 | 1.71 |
| Physical condition(s) | 1.18 | 1.31 | 1.15 | 1.36 |  | 0.92 | 1.18 | 1.07 | 1.61 | 1.34 | 1.30 |
| Female (vs male) | 1.28 | 1.00 | 0.96 | 0.72 |  | 1.99 | 1.32 | 1.08 | 0.97 | 0.90 | 0.97 |
| Average income (vs above-average) | 1.12 | 1.38 | 0.80 | 0.93 |  | 1.45 | 0.72 | 1.21 | 1.05 | 1.11 | 1.75 |
| Below-average income | 1.80 | 1.98 | 1.26 | 1.73 |  | 2.82 | 1.27 | 1.50 | 1.76 | 0.80 | 2.55 |
| Not born in country (vs born in country) | 1.69 | 1.10 | 1.18 | 0.87 |  | 1.72 | 1.31 | 1.63 | 1.56 | 0.74 | 1.27 |
| **Multiple barriers after reaching PC** | | | | | |  |  |  |  |  |  |
| 35 to 49 years (vs 18 to 34) | 1.06 | 0.85 | 1.07 | 1.32 |  | 0.63 | 1.49 | 0.99 | 1.12 | 0.69 | 0.72 |
| 50 to 64 years | 1.04 | 0.59 | 1.39 | 1.54 |  | 0.99 | 1.22 | 0.69 | 0.59 | 0.59 | 0.80 |
| 65 years and over | 0.33 | 0.43 | 1.15 | 1.89 |  | 0.55 | 0.90 | 0.41 | 0.38 | 0.60 | 0.35 |
| Mental health condition (vs no condition) | 2.04 | 1.57 | 0.74 | 0.78 |  | 1.46 | 1.65 | 1.98 | 0.84 | 1.48 | 1.00 |
| Physical condition(s) | 0.70 | 1.07 | 1.33 | 0.81 |  | 0.94 | 1.08 | 1.38 | 1.17 | 1.72 | 0.94 |
| Female (vs male) | 1.22 | 1.02 | 1.49 | 0.89 |  | 1.61 | 1.35 | 1.39 | 0.79 | 0.81 | 0.93 |
| Average income (vs above-average) | 1.51 | 1.48 | 1.92 | 1.09 |  | 0.74 | 0.98 | 0.97 | 1.01 | 1.05 | 1.46 |
| Below-average income | 1.38 | 1.82 | 3.32 | 2.58 |  | 1.74 | 1.48 | 1.22 | 1.47 | 1.19 | 2.03 |
| Not born in country (vs born in country) | 1.79 | 1.72 | 3.12 | 1.13 |  | 0.94 | 1.72 | 1.35 | 1.59 | 1.63 | 1.88 |

Note: *Darker shading indicates where all countries AOR are above 1, and lighted where AOR<1 (p<0.05). Missing values for countries indicate there were not sufficent responses with the barrier to access in that country to run the full model (n<100). Results are from full models within countries for each access barrier adjusting for the variables listed as well as hospitalisation in the past two years.*
